# Historical Perspective and Clinical Implementation of Pharmacogenomics (PGx) Technology with Clinical Decision Support (CDS) in Child and Adolescent Psychiatry

**DOI:** 10.3390/ph19101554

**Published:** 2026-10-01

**Authors:** Rachael Cella, Helen Bian, Alexander Luo, Bushra Alfakhri, Shanzay Malik, Salma Malik, Gualberto Ruaño

**Affiliations:** 1 University of Connecticut School of Medicine, 263 Farmington Avenue, Farmington, CT 06030, USA; 2 Department of Psychiatry, University of Connecticut Health Center, 263 Farmington Avenue, Farmington, CT 06030, USAsalma.malik@hhchealth.org (S.M.); gualberto.ruano@hhchealth.org (G.R.); 3 Department of Psychiatry, Eastern Connecticut Health Network, 71 Haynes Street, Manchester, CT 06040, USA; 4 Mercy High School, 1740 Randolph Road, Middletown, CT 06457, USA; shanzayzmalik2008@gmail.com; 5 Institute of Living at Hartford Hospital, 200 Retreat Avenue, Hartford, CT 06032, USA

**Keywords:** pharmacogenomics, child and adolescent psychiatry, psychotropics, cytochrome P450, clinical decision support, personalized medicine

## Abstract

Pharmacogenomics (PGx) uses the variation in a patient’s genetic profile to assist with medication choice, with the goal of shortening the trial-and-error process of prescribing in psychiatry. Over the past few decades, PGx technology has evolved greatly to identify various genetic polymorphisms in drug-metabolizing enzymes; however, clinical use and relevance of PGx in child and adolescent psychiatry (CAAP) remain limited. This narrative review explores the historical evolution of PGx, compares key technological advancements, and evaluates their clinical relevance to CAAP. We address current barriers to PGx implementation, including developmental differences in pharmacokinetics between adults and children, the lack of robust evidence of clinical efficacy in youth populations, and inconsistent guidelines causing clinician confusion. We also highlight the need for clinical decision support (CDS) tools and broader practical and ethical considerations before widespread uptake of PGx would be feasible.

## 1. Introduction

Pharmacogenetics, how variation in a single gene influences drug metabolism and response, has broadened over recent decades into pharmacogenomics (PGx)—the study of how genome-wide variation affects a person’s response to medications. Theoretically, PGx allows physicians to personalize medication therapy to each patient based on their unique genetic profile. This is especially relevant to the field of psychiatry, since treatment tolerability and efficacy are highly heterogeneous between patients, typically requiring a long trial-and-error process to optimize clinical outcomes [1,2]. Its potential is especially relevant to child and adolescent psychiatry (CAAP) because youth are particularly vulnerable to medication side effects due to ongoing changes in drug metabolism during development. It also may be useful for treatment-resistant patients, those who have experienced adverse side effects, or those who have significant comorbidities that may worsen with certain medications.

In psychiatry, PGx testing mostly focuses on pharmacokinetic genes, such as those encoding cytochrome P450 enzymes, since they metabolize most antidepressants, stimulants, and antipsychotics [1,3]. Polymorphisms in these genes are responsible for ultrarapid or poor metabolism, thus altering drug levels in the plasma and increasing the risk of inefficacy or toxicity, respectively.

## 2. Historical Evolution of PGx

The field of pharmacogenetics emerged in the 1950s after scientists discovered that genetic differences could alter an individual’s response to a drug (Figure 1). In 1959, Friedrich Vogel coined the term “pharmacogenetics,” and in 1962, Werner Kalow published the first textbook dedicated to the field. Subsequent discoveries of enzymatic polymorphisms in clinically relevant genes (such as CYP2D6) were made with the later standardization of CYP450 allele nomenclature, making it easier for experts to understand genetic variation in drug metabolism. With the launch of the Human Genome Project in 1990 and its completion in 2003, the ability to discover multiple genes using high-throughput technologies accelerated rapidly, thus creating the field of PGx. Throughout this decade of innovation, organizations such as the Pharmacogenomics Knowledgebase (PharmGKB) and the Pharmacogenetics Research Network (PGRN) were created to help collect, organize, and assist with interpretation of a large amount of new PGx data, and Dr. David Flockhart published the widely used CYP450 drug interaction table. Soon thereafter, in 2004, the FDA cleared the first pharmacogenetic test known as the AmpliChip CYP450 test, which focused on using microarray technology to identify polymorphisms in CYP2D6 and CYP2C19. The subsequent establishment of the Dutch Pharmacogenetics Working Group (DPWG) in 2005 and the Clinical Pharmacogenetics Implementation Consortium (CPIC) in 2009 aimed to help clinicians translate PGx evidence into real-world prescribing decisions. More recently, the creation of PharmVar in 2017 and the FDA’s publication of the Table of Pharmacogenetic Associations in 2020 further advanced standardization of nomenclature and clinical implementation.

Although new methods of investigating the human genome were developed and available, such as next-generation sequencing, most laboratories still use targeted genotyping panels due to lower costs and faster turnaround times [3,4].

## 3. Technological Advances in PGx Testing

### 3.1. DNA Microarrays

The use of single-nucleotide variant (SNV) panel testing in DNA microarray technology has revolutionized modern-day PGx. Also known as DNA chips or GeneChips, microarrays are the primary genotyping technology in PGx today [4]. If a variant is present, it binds to the complementary probe and generates a fluorescent signal when passed under a laser, allowing for massive parallel genotyping [5].

The AmpliChip CYP450 test (Roche Diagnostics) was the first FDA-cleared microarray for pharmacogenetic testing [6]. It simultaneously analyzed 27 CYP2D6 alleles (including seven gene duplications) and 3 CYP2C19 alleles [7]. Individuals were classified into two CYP2C19 phenotypes (extensive or poor metabolizers) and four CYP2D6 phenotypes (ultrarapid, extensive, intermediate, or poor metabolizers), each corresponding to enzyme activity levels [7].

Sample collection methods, including buccal swabs, saliva, or blood, are determined by the laboratory’s validated workflow and are not unique to a particular genotyping technology. DNA microarrays are fixed and inflexible; after development of a chip, an entirely new design is required to accommodate additional genes or variants of interest (Table 1). A microarray can identify a rare allele only when that allele is represented by a probe on the array; it cannot discover novel variants or interrogate genomic regions that were not included in the panel. Some complex structural variants and copy-number changes may also be difficult to resolve, depending on assay design and the pharmacogene of interest.

### 3.2. Bead-Based Microarrays

The bead-based microarray is very similar to the DNA microarray but utilizes micro-spheres (referred to as “beads”) instead of fixed DNA “spots” on a chip to detect SNPs. Once hybridization occurs between the patient’s DNA and the beads, the beads emit a fluorescent signal that is transmitted to the detection system [8]. This contrasts with the traditional microarray, which has predetermined spots of probes printed onto a slide (Table 1).

The Infinium™ Global Diversity Array with Enhanced PGx-8 v1.0 BeadChip covers over 6000 variants from PGx databases, including CYP2D6 [9].

### 3.3. Mass Spectrometry

Mass spectrometry (MS) is a technique that identifies and quantifies molecules based on their mass-to-charge ratio (*m*/*z*) [10]. In this approach, a laser irradiates the sample, ionizes the analyte without significant fragmentation, and accelerates the ions towards the detector [10]. Heavier ions take longer to arrive (longer time of flight) than lighter ones [11]. The Agena Bioscience MassARRAY^®^ System is a mass spectrometry platform that genotypes tens to hundreds of pre-selected SNPs simultaneously [12].

### 3.4. Next-Generation Sequencing

NGS produces substantial data that may require more extensive analysis and interpretation (Table 1). Cost and turnaround time vary by laboratory, assay scope, workflow, and clinical setting.

## 4. Clinical Relevance of PGx in Child and Adolescent Psychiatry

The GUIDED trial, a randomized controlled trial of over 1100 adults with major depressive disorder and prior treatment failure [13], did not show a statistically significant difference in the primary outcome of overall symptom improvement in the full cohort. However, PGx-guided care was associated with significantly higher rates of treatment response and remission. In a post hoc analysis accounting for patients already receiving medication congruent with their PGx report at baseline, symptom improvement also became statistically significant. These findings suggest potential value in selected adult populations but do not establish clinical utility in children and adolescents.

In youth, clinical outcomes like faster symptom relief and fewer side effects due to PGx carry even more weight because of their ongoing development and the need for safe long-term treatment; however, studies are lacking. A recent randomized controlled trial examining PGx-guided antidepressant prescribing in adolescents showed no significant differences in symptom improvement or side effect burden compared to standard care [14].

Application of adult PGx guidance in pediatric populations is difficult due to fundamental differences in pharmacokinetics between children and adults. Studies on SSRIs in kids have shown that pediatric variation in pharmacokinetic genes influences drug plasma concentration and drug tolerability, but differences in drug exposure do not consistently predict clinical response or adverse effects [15]. In addition, phase I metabolism is often faster in children, with body-weight-normalized drug clearance being up to 2-4-fold higher in kids than in adults, increasing the risk of subtherapeutic drug exposure even among genotypically normal metabolizers [1].

### Pediatric Dosing Frameworks Informed by PGx

Pharmacokinetic modeling studies have examined patients with known CYP2C19 genotypes and plasma SSRI concentrations, demonstrating that those classified as poor metabolizers were overtreated (had supratherapeutic SSRI levels when given standard SSRI doses), whereas those classified as ultrarapid metabolizers were undertreated (had subtherapeutic SSRI levels when given standard SSRI doses). They noted that differences in plasma concentration were larger than what could be attributed to developmental age alone. This study shows how PGx can inform SSRI dosing in children [16]. Currently, only some SSRIs have actionable dosing guidance for different CYP2C19 genotypes, including citalopram, escitalopram, and sertraline. Several antipsychotics (metabolized by CYP2D6) and atomoxetine (metabolized by CYP2D6) also include actionable guidance. However, many drugs still require more thorough investigation (such as stimulants). Research has shown that children who are poor metabolizers of atomoxetine due to CYP2D6 variants tend to exhibit a greater reduction in ADHD symptoms, likely because less efficient metabolism leads to higher plasma drug levels [17].

## 5. Barriers to Clinical Implementation of PGx

### 5.1. Insufficient Evidence of Clinical Utility in Pediatrics

There is limited evidence to support the use of PGx in CAAP at this time, creating one of the largest barriers to widespread implementation. The American Academy of Child and Adolescent Psychiatry (AACAP) recommends avoiding the use of PGx to guide prescribing [18]. The American Psychiatric Association (APA) has a similar perspective, as it also has not formally endorsed routine PGx use [19]. While some trials suggest that PGx-guided prescribing may improve treatment outcomes such as remission rates, much of this data comes from studies that are commercially affiliated, raising concerns about potential bias and generalizability [20].

Theoretical models and biological plausibility suggest that therapeutic drug monitoring (TDM) may be beneficial when prescribing psychiatric medications to youth. Although adult-derived therapeutic ranges may provide some preliminary guidance, SSRI exposure in children is not determined by CYP genotype alone. Drug concentrations are influenced by multiple confounding factors, including developmentally regulated enzyme activity, drug–drug interactions resulting in phenoconversion, and pharmacodynamic genetic variants such as SLC6A4 [15]. Prospective trials are needed to determine whether TDM-guided dosing frameworks improve medication efficacy and tolerability in children and adolescents [1].

### 5.2. Developmental Considerations

Another impediment to PGx implementation in CAAP is the variability in drug metabolism and response among youth. Developmental differences in pharmacokinetics between children and adults, including altered distribution volumes and age-dependent drug clearance, further complicate the applicability and interpretation of PGx results in pediatric populations. Pharmacokinetic enzyme activity rapidly changes throughout childhood and adolescence, making predictions of metabolizer phenotypes from genotypes difficult, especially since most data comes from adult studies [1,21]. As a result, metabolizer status based on genotype alone may inadequately reflect functional enzyme activity at different developmental stages.

Therefore, genotype–phenotype translational frameworks used in adults do not consistently predict drug exposure or clinical response in pediatric patients. Children with identical metabolizer phenotypes may require substantially different dosing to achieve therapeutic drug concentrations due to age-related factors [22]. Additionally, CYP2C19 status in youth treated with escitalopram or citalopram has been linked to tolerability and time to response, but not consistently with treatment efficacy, differing from adult studies [23].

### 5.3. Uncertain Cost-Effectiveness and Financial Barriers

High testing costs and inconsistent insurance reimbursement significantly limit access and clinician uptake. After performing an analysis of insurance claims for PGx testing coverage, Lemke et al. [24] found that the overall reimbursement rate was about 48% but depended heavily on the type of test ordered, with multigene panels significantly more likely to be covered than single-gene tests.

Insurance companies frequently have different evidentiary standards on what is adequate to support coverage of a particular test, with some citing society guidelines, others citing randomized controlled trials, and others following regulatory agencies. These barriers likely dissuade providers from ordering PGx testing to avoid burdensome paperwork required to deal with insurance denials. Additionally, it is uncertain which type of patient should receive PGx testing, with some recommending it preemptively while others suggest reactive testing [25].

### 5.4. Clinician Knowledge Gaps

Many clinicians still do not feel confident using PGx in practice, and that is a big part of why it has not caught on more widely. In one national survey, nearly half of child and adolescent psychiatrists said their knowledge of PGx and the related guidelines was “poor” or “very poor” [19]. Other studies have shown a similar sentiment.

In a survey of almost 600 psychiatric patients, most people said they were interested in PGx, but barely any had heard about it from a doctor [26]. So even when patients are curious and open about treatment decisions, they are not getting information from the people they trust most.

### 5.5. Lack of Guidelines, Standardization, and Regulatory Alignment

One of the reasons many clinicians are hesitant to use PGx is that the guidelines are not consistent. Comparative studies have demonstrated significant variability in medication recommendations across commercial PGx platforms, even when genotyping the same variants [27]. Additionally, regulatory oversight in the U.S. remains fragmented. Most PGx tests are classified as lab-developed tests (LDTs), which means they are subject to Clinical Laboratory Improvement Amendments (CLIA) but are not tightly regulated by the FDA [19]. Although CLIA may attempt to ensure that the analytical performance of LDTs is validated, it does not evaluate the clinical utility of these tests like the FDA would, making it impossible to provide uniform assurance of LDTs.

Lack of consistency in clinician guidance is not just limited to commercial PGx test results. Shugg et al. [28] found that about 48% of PGx recommendations varied between CPIC, the FDA, and professional medical organizations. For gene–drug pairs that were addressed by all three sources, over 90% contained at least one inconsistency. This discordance creates uncertainty for clinicians and a barrier to reimbursement.

Although FDA-approved labeling increasingly acknowledges the influence of pharmacokinetic genes on drug metabolism, dosing recommendations are largely derived from adult populations, limiting their applicability to pediatrics [29]. Adolescent modeling studies suggest that doses exceeding FDA-approved pediatric maximums may be required to achieve therapeutic drug exposure in certain metabolizer groups, further highlighting these limitations [16].

In contrast, guidelines developed by organizations such as the Clinical Pharmacogenetics Implementation Consortium (CPIC) and the Dutch Pharmacogenetics Working Group (DPWG) are systematically evidence-graded and designed to support genotype-informed prescribing. However, these recommendations frequently diverge from FDA guidance, contributing to clinical uncertainty [30]. For example, FDA labeling for atomoxetine primarily relies on weight-based dosing despite recognizing CYP2D6 metabolism, whereas CPIC recommends genotype-informed dose escalation guided by TDM in patients with an inadequate response [22]. Because insurers typically prioritize FDA-approved labeling, such discrepancies may also affect reimbursement.

The inclusion of PGx-relevant information on drug labels regulated by the FDA is different from the recommendations made by CPIC. The FDA focuses mainly on the existence of evidence and avoids giving clinicians any guidance in prescribing, while CPIC stresses clinically actionable recommendations to implement when treating patients that directly link evidence quality to the importance of each recommendation [31].

### 5.6. Antidepressants with Conflicting Recommendations

A comparative evaluation of PGx-informed recommendations for commonly prescribed SSRIs made by the FDA, CPIC, and DPWG reveals many discrepancies, creating confusion about which guidance to follow [31]. The same gene–drug pairs may be deemed actionable by one regulatory body or expert consortium but not another, underscoring the lack of harmonization [32]. Collectively, these discrepancies illustrate inconsistent guidance across major regulatory bodies and professional agencies, contributing to uncertainty in clinical decision-making, especially in pediatric populations who are frequently prescribed medications with actionable CYP2D6 and CYP2C19 PGx guidance [33].

### 5.7. Workflow Integration and Infrastructure Limitations

PGx testing often introduces logistical disruptions into clinical workflows. Without integrated electronic health record (EHR) systems and clinical decision support (CDS), the results may be difficult to access, interpret, or apply when time is of the essence. For example, a feasibility study performed in a CAAP clinic illustrated that PGx results took about 10 days to return after the sample was obtained, with an additional 5 days before the patient’s genotype was translated into an actionable phenotype that the physician could use to make treatment decisions [34].

### 5.8. Lack of Guidance on Appropriate Timing of PGx Testing

There is an absence of agreement on when to use PGx testing during a patient’s treatment course (i.e., before, during, or after), and there is uncertainty on how much the result may align with the patient’s clinical status. Some clinicians may be inclined to use PGx testing to confirm an adverse effect or treatment failure; however, it has been shown that reactive testing as such can only explain a small fraction of clinical events or responses [35]. The consequence of this gap is illustrated by Soda et al. [19], who found that some providers changed their patients’ medication regimens based on PGx results, even when patients were stable on their current medication and side effect-free. These findings underscore that PGx results should not replace clinical judgment.

### 5.9. Exaggeration in Media and Unrealistic Patient Expectations

The expectations of both patients and families further influence implementation of PGx in psychiatry. Unfortunately, when the efficacy of PGx is exaggerated to laypeople, it may promote unrealistic expectations amongst patients. Soda et al. [19] found that 62.8% of child and adolescent psychiatrists had been asked by a family to order PGx testing within the last year, and more than 40% of psychiatrists agreed, even when there was no clinical indication to order the test.

## 6. Clinical Decision Support (CDS)

Deploying PGx data in clinical practice is very challenging. Clinicians often report that applying PGx results to a prescribing decision in clinical practice is a significant challenge. “CDS tools” refers to healthcare information presented through technological modalities to key stakeholders (e.g., pharmacists, physicians, nurses, and patients) within the clinical workflow. A widely accepted approach to optimizing CDS is adherence to the “Five Rights” framework, which is not specific to PGx but applies broadly to the delivery of clinical information in general (i.e., lab results, medication-safety alerts, diagnostic information, and prescribing guidance). This framework emphasizes delivering the right information to the right person, using the right intervention format, through the right channel, at the right time in the clinical workflow [36]. When using the five rights framework to optimize CDS for PGx results, it is important that raw genotypes be translated into a predicted phenotype before the information can be understood and become clinically actionable. It also requires an easily accessible place for storage within the EHR and the ability to time delivery of information properly when making decisions on appropriate drug choice and dosing (Table 2).

ClinPGx is a comprehensive clinical PGx resource created to support and expand PGx knowledge, implementation and education. Users can search the site for specific drugs or gene variants to aid in providing supplemental prescribing guidance on effective dosing.

Sequence2Script is an online resource that collects a variety of information about your patient, such as their genetic information, their current medications, and the medications the clinician is considering using. After collecting that data, it uses a multitude of outside resources such as CPIC, DPWG, FDA, PharmVar, PharmGKB, and the Flockhart table to compile an evidence-based recommendation for medication and dosing.

While some analyses suggest long-term savings from reduced hospitalizations and more efficient prescribing [37], the upfront costs for implementing robust CDS infrastructure pose a significant challenge.

## 7. Ethical and Practical Considerations

### 7.1. Incidental Findings

The likelihood of incidental or secondary findings depends strongly on the scope of the PGx test. Most PGx assays used in psychiatric practice are targeted panels or microarrays that interrogate predefined variants in selected pharmacogenes. Because these assays are designed to answer a limited medication-related question, they generally have little potential to identify unrelated genetic findings. In contrast, broader sequencing approaches, including expanded multigene panels, exome sequencing, and whole-genome sequencing, may identify variants outside the immediate PGx indication. When broad sequencing is used, clinicians should establish in advance whether and how secondary findings will be analyzed, reported, and discussed with families.

### 7.2. Informed Consent

In CAAP, additional ethical considerations arise when obtaining informed consent due to the need to balance consent from parents with assent from children.

For young children (under 7 years old), a simple, concrete explanation of the sample collection process is sufficient, as they are not yet able to grasp the risks, benefits, and long-term implications of testing [38]. For older children and adolescents who can understand the implications of testing, the physician should ask for their assent, as it demonstrates respect for their growing autonomy and helps them develop decision-making skills.

The amount and type of information disclosed during the informed consent process depends on the PGx testing modality. For example, when using targeted PGx panels that examine a predefined set of genes, these results carry fewer long-term implications because they serve to inform medication selection and dosing rather than establishing a diagnosis or predicting risk of disease. It is important to note that scientific evidence may change over time, possibly altering future interpretation of results. When using expanded panels or whole-genome sequencing, the disclosure should be broader and include discussions surrounding maintenance of genetic privacy, secondary genetic findings, and proper storage of results. Therefore, the informed-consent discussion should be tailored to the scope of testing.

### 7.3. Iatrogenic Effects of Psychotropics

If a clinician solely depends on PGx test results when making treatment decisions, they may ignore other important considerations, such as a patient’s prior response to treatment, their comorbidities, or even their medication preference, thus inadvertently resulting in iatrogenic harm.

The United States Food and Drug Administration (FDA) has warned against using unapproved genetic tests that lack validation to predict medication response, emphasizing the potential for serious health consequences if medication regimens are changed solely based on PGx results [39]. They should never replace clinical and environmental considerations, such as drug–drug interactions and patient adherence, as genetic variations are only one factor affecting medication response.

### 7.4. Diversity of Global Populations

Most of the existing PGx research has disproportionately been performed using North American and European populations, without adequate representation of others. Over 78% of participants in genome-wide association studies (GWAS) are of European descent, with 71.8% recruited from just three countries: the United States, the United Kingdom, and Iceland [40]. International PGx studies across diverse populations may reveal genetic variations across different ethnicities that influence an individual’s metabolic phenotype and possible response to medication [41,42].

## Figures and Tables

**Figure 1:**
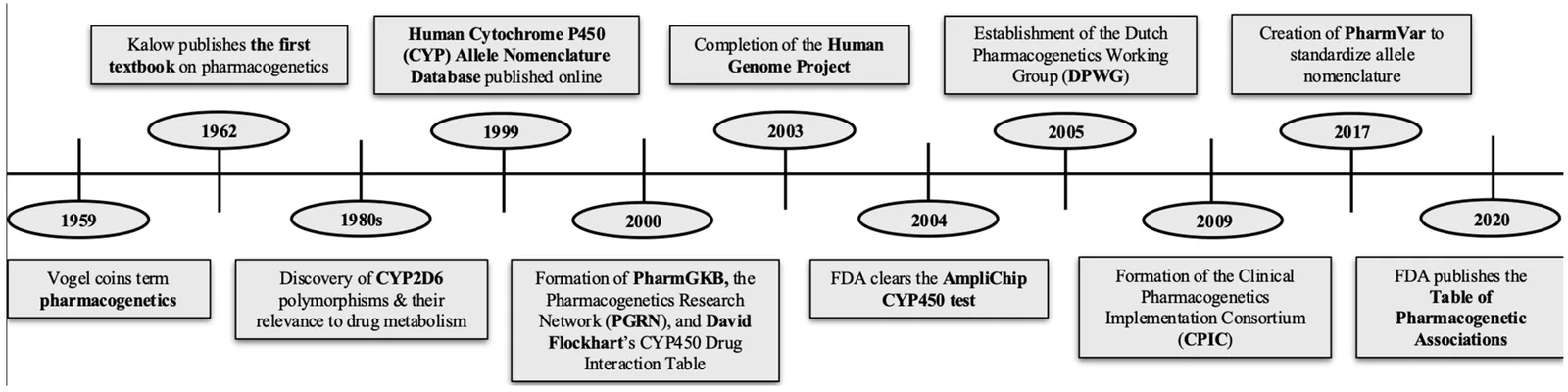
Timeline of major developments in pharmacogenomics.

**Table 1:** Advantages and limitations of PGx technologies.

Technology	Advantages	Limitations
DNA Microarray	Rapid, high-throughput genotyping Efficient targeted analysis of predefined multigene content Established clinical utility for common PGx variants Compatible with laboratory-validated collection methods Widely available	Inflexible, making it difficult to update panels as new variants are discovered Limited to known variants (cannot detect new or rare polymorphisms) Results may be affected by DNA quality, cross-hybridization, or background noise Struggles to detect complex structural variations
Bead-Based Microarray	Flexible design allows customization of PGx panels High multiplexing capacity	May involve platform-specific DNA preparation, including amplification, fragmentation, or whole-genome amplification Cannot detect novel variants or variants not represented in the assay design Additional steps like decoding add time and complexity
Mass Spectrometry (MS)	Highly accurate & precise for targeted genotyping Straightforward data interpretation	Requires expensive specialized equipment & technical expertise Lower throughput for large-scale panels Limited in detecting structural changes or large copy-number variations
Next-Generation Sequencing (NGS)	Comprehensive detection of both common & rare variants Applicable across diverse populations Potential for discovery of new polymorphisms and structural variants	Resource requirements vary by assay scope, laboratory workflow, and clinical setting Turnaround time varies by assay scope and laboratory workflow Interpretive complexity Challenges with sequencing certain pharmacogenes (e.g., CYP2D6 pseudogene interference) Variants of uncertain significance (VUS) complicate clinical utility Reimbursement varies by payer policy, test, and clinical indication

This table lists the advantages and limitations of the core technologies used in pharmacogenomic testing.

**Table 2:** Application of the general five rights clinical decision-support framework to pharmacogenomic information.

Five Rights	Meaning	PGx-Specific Implementation
Right information	Clinically relevant, evidence-based, actionable guidance	Provide clinicians with recommendations about dosing or drug choice based on metabolizer phenotype rather than reporting raw genotype data
Right person	Delivered directly to the prescribing psychiatrist making the treatment decision	Send the result to the prescriber of the affected drug, which may be different due to transitions or availability of care
Right intervention format	Presented in a clear, usable format (e.g., dosing recommendation in a note, not buried in a PDF)	Pop-up warnings only in high-risk situations (e.g., prescribing a high-dose psychotropic medication to a poor metabolizer) to avoid alert fatigue
Right channel	Delivered through the correct system (e.g., inside the EHR)	Enter PGx results into the electronic health record (rather than simply scanning) so they are easily accessible
Right time in workflow	Available exactly when the clinical decision is being made	Guidance arises automatically in the EHR when a psychotropic medication is ordered to inform drug choice and dosing at the moment of prescribing

## Data Availability

No new data were created or analyzed in this study. Data sharing is not applicable to this article.
